# TGF-β signaling mediates crosstalk between CD8^+^ T cells and CD39^+^ induced Treg cells in autoimmune inflammation

**DOI:** 10.1186/s12929-026-01228-z

**Published:** 2026-02-24

**Authors:** Ye Chen, Jun Long Dang, Qi Long, Nianke Zang, Ling Liu, Yang Lu, Wei Zhao, Rong Zhen Liang, Yi Yang, Jun Zhao, Jing Rong Chen, Yi Ding Xiong, Julie Wang, Yun Feng Pan, Nancy Olsen, Song Guo Zheng

**Affiliations:** 1 Division of Rheumatology and Immunology, Department of Internal Medicine, Shanghai Songjiang Hospital Affiliated to the Shanghai Jiao Tong University School of Medicine, Shanghai, 201600 China; 2 Department of Immunology, School of Cell and Gene Therapy, Songjiang Research Institute, Shanghai Songjiang Hospital Affiliated to the Shanghai Jiao Tong University School of Medicine, Shanghai, 201600 China; 3 State Key Lab of Innovative Immunotherapy, Shanghai Jiao Tong University, Shanghai, 201600 China; 4 Division of Rheumatology, Department of Internal Medicine, The Third Affiliated Hospital of Sun Yat-Sen University, Guangzhou, 510000 China; 5 The First Dongguan Affiliated Hospital, Guangdong Provincial Key Laboratory of Medical Molecular Diagnostics, School of Medical Technology, State Key Laboratory of Pathogenesis, Guangdong Medical University, Dongguan, 523710 China; 6 Department of Endocrinology and Metabolic Diseases, The Third Affiliated Hospital of Sun Yat-Sen University, Guangzhou, 510630 Guangdong China; 7 Division of Rheumatology, Penn State College of Medicine and Milton S. Hershey Medical Center, Hershey, PA 17033 USA

**Keywords:** CD8^+^ T cells, Induced Treg cells, Autoimmune inflammation, ROS, TGF-β, IRF4

## Abstract

**Background:**

Autoimmune inflammation results from dysregulated immune responses, with dysfunction of regulatory T cells (Tregs) being a key contributor due to their critical role in maintaining immune tolerance. The stability and function of Tregs are strongly influenced by the inflammatory microenvironment, yet the regulatory interactions between CD8^+^ T cells and CD4^+^Foxp3^+^ Tregs remain poorly understood.

**Methods:**

We investigated the role of CD8^+^ T cells in regulating induced Tregs (iTregs) using in vitro T cell co-culture assays and two in vivo models of autoimmune disease. A naïve CD4^+^ T cell transfer colitis model was used to evaluate the suppressive function of iTregs in the presence or absence of CD8^+^ T cells, while an autoimmune arthritis model was employed to assess therapeutic efficacy. Flow cytometry, functional suppression assays, and mechanistic analyses were performed to define signaling pathways.

**Results:**

CD8^+^ T cells promoted the differentiation of a CD39^+^ iTreg subset characterized by increased frequencies of CD103, CTLA-4, and Helios, leading to enhanced immunosuppressive capacity. In the colitis model, co-transfer of CD8^+^ naïve T cells alleviated disease by reinforcing iTreg-mediated suppression of Th1 and Th17 responses. Mechanistic studies revealed that CD8^+^ T cells regulated iTreg phenotype through a ROS/TGF-β signaling axis, with IRF4 in CD8^+^ T cells acting as a key mediator. Importantly, CD8^+^ T cell–primed iTregs showed superior therapeutic efficacy in the autoimmune arthritis model by suppressing pathogenic Th1/Th17 responses and supporting endogenous Treg homeostasis.

**Conclusions:**

This study identifies a previously unrecognized role of CD8^+^ T cells in enhancing iTreg differentiation, stability, and suppressive function through the ROS/TGF-β–IRF4 pathway. These findings reveal a novel mechanism of immune regulation and suggest that harnessing CD8^+^ T cell–primed iTregs could represent a promising strategy to strengthen Treg-based therapies for autoimmune diseases.

**Supplementary Information:**

The online version contains supplementary material available at 10.1186/s12929-026-01228-z.

## Introduction

Autoimmune diseases, such as inflammatory bowel disease (IBD) and rheumatoid arthritis (RA) are characterized by the dysregulation of immune system [1]. CD4^+^ T helper (Th) cells are well recognized as a key driver that results in the tissue destruction in many inflammatory and autoimmune diseases, including IBD and RA [2]. Among the Th cells, pathogenic Th1/Th17 cells can facilitate inflammatory responses by producing IFN-γ and IL-17A, and to initiate and persist the pathogenesis of IBD and RA [3, 4]. So far, the major therapeutics for IBD and RA are nonsteroidal anti-inflammatory drugs (NSAIDs) and conventional biologic disease-modifying antirheumatic drugs (DMARDs). However, not all patients respond to these available treatments [5]. Therefore, novel effective therapies need to be developed for these autoimmune diseases.

Regulatory T cells (Treg, CD4^+^ Foxp3^+^) are an immunosuppressive subset of CD4^+^ T cells capable in controlling inflammation in diverse settings [6]. Treg cells prevent autoimmune response and maintain immune homeostasis via multiple approaches and mechanisms, including CTLA-4, CD39, CD73, IDO, TGF-β, IL-10, TIGIT and others [7–9]. Deficiency of Foxp3 or functional molecules above in Treg cells can indeed result in dysregulation of immune system and facilitate the development of autoimmune disorders [10]. The indispensable role of Tregs in various autoimmune diseases has opened a new therapeutic avenue. Thus, targeting Treg cell differentiation or therapies aiming to restore the balance of Treg and Teff cells (IL-2 therapies) can definitely alleviate the development of autoimmune diseases [11, 12].

We previously documented that TGF-β-induced Treg cells are effective in treating various inflammatory dysregulation associated diseases, such as autoimmune arthritis [13–16], lupus-like disease [17–19], GVHD [20], T1D [21], et al. However, the complex microenvironment in autoimmune disorders may exert multiple effects on Treg cell targeted therapy. Interestingly, CD39 is an ectoenzyme that hydrolyzes adenosine triphosphate (ATP) and adenosine diphosphate (ADP) into adenosine monophosphate (AMP). Our previous study demonstrated that CD39^High^ Tregs exhibit greater stability and enhanced immunosuppressive function [22]. However, whether regulating CD39 can rheostat Treg cell stability as well as the underlying mechanism remain unknown.

The susceptibilities of autoimmune diseases have strong links to major histocompatibility complex (MHC) loci, class I or class II [23]. CD8^+^ T cells show tight link to organ-specific autoimmune diseases [24]. For instance, CD8^+^ T cells directly correlate to autoimmune arthritis disease severity and outcome [24]. The autoimmune stem-like CD8^+^ T cells are considered to drive type 1 diabetes [25]. In addition, tissue resident CD8^+^ T cells are essential to drive chronic CNS autoimmunity and crucially dependent on CD4^+^ T cells [26]. Furthermore, CD8^+^ T cells are capable in governing SLE pathogenesis [27]. Thus, CD8^+^ T cells may play a broad-spectrum role in modulating autoimmune diseases. Importantly, CD4^+^ Treg cells can indeed govern CD8^+^ T cell phenotype and function in immune response [28]. However, whether CD8^+^ T cells can in turn determine CD4^+^ Treg cells suppressive capacity and stability, as well as the potential mechanism has never been uncovered.

In this study, we investigated the influence of CD8^+^ T cells on CD4^+^ iTreg induction and function. We explored how CD8^+^ T cells modulate iTreg stability, functional marker expression, and therapeutic efficacy in inflammatory disease models, including colitis and autoimmune arthritis. Our findings reveal a previously unrecognized role of CD8^+^ T cells in enhancing iTreg-mediated immune suppression through a ROS/TGF-β-dependent mechanism. Furthermore, we identified IRF4 as a critical transcription factor in CD8^+^ T cells that facilitates iTreg differentiation and function. By elucidating these novel interactions, our study provides important insights into Treg biology and offers a potential strategy for optimizing Treg-based therapies in autoimmune diseases.

## Results

### Selective enhancement of CD4^+^ iTreg function by CD8^+^ T cells in vitro

Induced regulatory T cells (iTregs) are a promising cell type for immunotherapy due to their potent capacity to suppress excessive immune responses and maintain immune tolerance. However, compared with natural Tregs (nTregs), iTregs often exhibit reduced stability of Foxp3 expression and impaired suppressive function under inflammatory conditions, limiting their clinical potential [29]. nTreg differentiation and maturation involve interactions with multiple immune cell types, prompting us to investigate whether other cell subsets influence iTreg induction.

To this end, we induced iTreg cells from either naïve CD4^+^ T cells or Foxp3-GFP^−^ spleenic cells. Although splenocyte-derived iTregs showed a slightly reduced proportion of Foxp3^+^ cells compared with those derived from purified naïve T cells, they expressed markedly higher levels of key functional molecules, including CD39, Helios, CTLA-4, and CD103(Fig. S1a and b). This observation suggests the possible involvement of additional cells in the spleen microenvironment that enhances the functional phenotype of iTregs.

We next sorted the major spleen cell subsets as shown in the Supplementary Fig. 1c, and co-cultured with naïve CD4^+^ T cells at a 1:1 ratio. The results showed that none of these subsets significantly promoted CD39 frequency in iTregs. Notably, CD11b^+^ monocytes were found to reduce Foxp3 percentage, while neither B220^+^ B cells nor CD44^+^ memory CD8^+^ T cells altered Foxp3 frequency (Fig. S1c-d). This phenomenon may also explain the reduced Foxp3 percentage observed in iTregs derived from splenic cells.

**Fig. 1:**
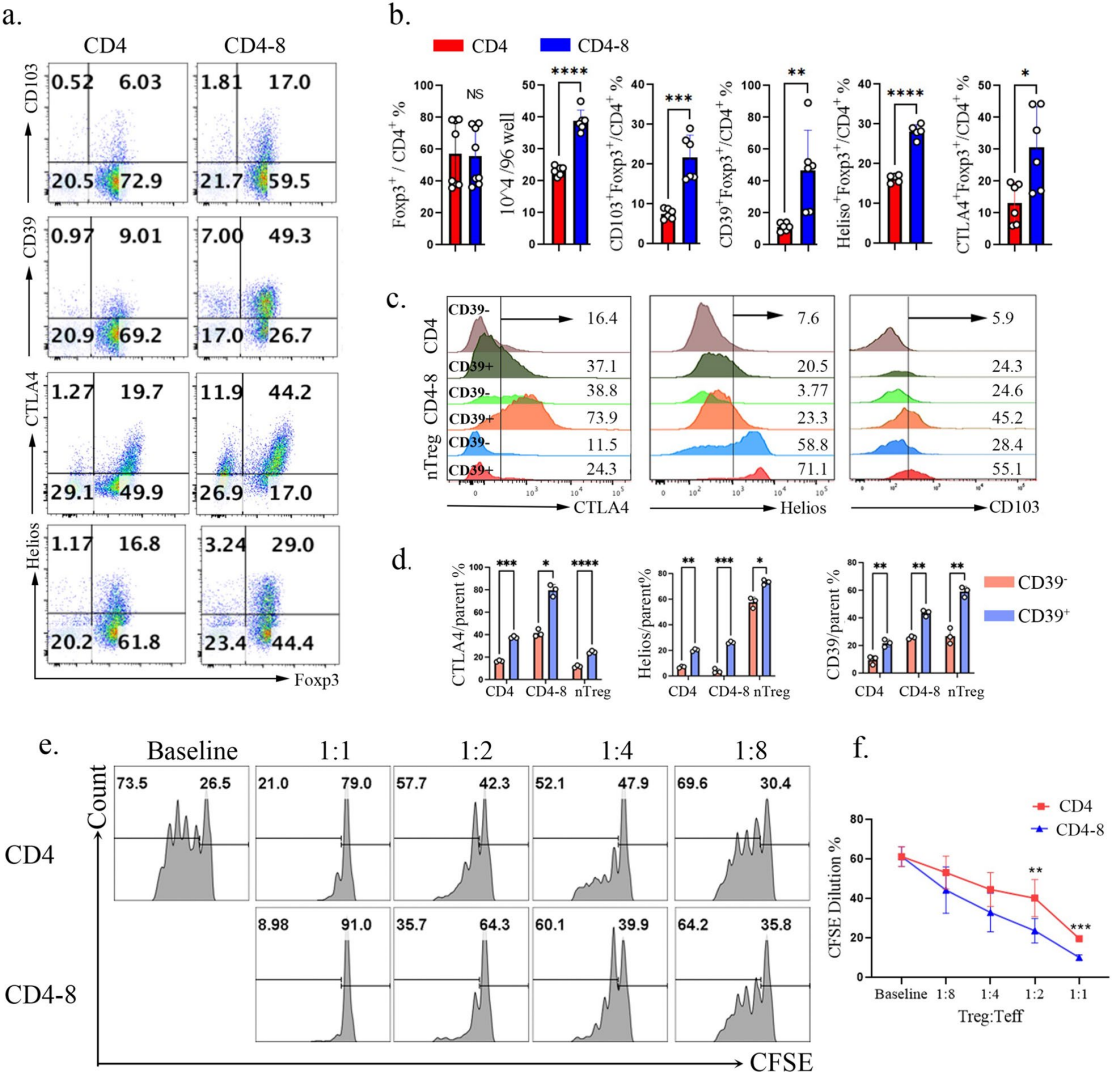
Selective enhancement of CD4^+^ iTreg function by CD8^+^ T cells in vitro. Naive CD4^+^ and CD8^+^ T cells were isolated from mouse spleen and the purity was above 95%. Naive CD45.2^+^ CD4^+^ T cells were polarized under iTreg conditions in the presence of equal numbers of naive CD45.1^+^ CD4^+^ or CD45.1^+^ CD8^+^ T cells. **a**, **b**. Flow cytometry was performed to assess the percentage of Foxp3, Helios, CTLA4, CD39 and CD103 in CD45.2^+^CD4^+^ cells, the representative flow data (**a**) and the statistical analyses(**b**) were shown; **c**, **d**. Flow cytometry was used to quantify the percentage of CTLA4, Helios, and CD103 in CD39^+^ and CD39^−^ Treg subsets, including both above in vitro-generated iTregs and in vivo derived nTregs, the representative flow data (**c**) and the statistical analyses(**d**) were presented; (**e**, **f**). CD4^+^Foxp3^+^ iTregs were purified from these two groups and the function was analyzed by T cell proliferation assay. Data are representative of three independent experiments. *p* values were calculated by Students’ t test (**b**, **d**) or oneway ANOVA (**f**) followed by Tukey’s multiple comparisons test. Data are presented as mean ± s.e.m.*p < 0.05, **p < 0.01, ***p < 0.001, ****p < 0.0001,ns, not significant

Interestingly, only naïve CD8^+^ T cells significantly increased the frequencies of CD39^+^, Helios^+^, CTLA-4^+^, and CD103^+^ cells within the iTreg cells (Fig. 1a and b**).** Although they did not significantly alter the frequency of Foxp3^+^ cells, they markedly increased the absolute number of Foxp3^+^ cells (Fig. 1b**).** CD39, by mediating adenosine production, plays a crucial role in Treg suppressive function, and our previous data indicated that human CD39^high^ Tregs exhibit greater stability and in vivo functionality [22]. Consistently, we found that CD39^high^ Tregs—whether derived from naïve CD4^+^ T cells, CD4^+^/CD8^+^ co-cultures, or nTregs in vivo—displayed higher frequencies of CTLA-4^+^, Helios^+^, and CD103^+^ cells (Fig. 1c and d). These findings suggest that CD39 may serve as a key functional marker of Tregs. Accordingly, we selected CD39 as one of the representative functional molecules for further characterization CD4-8 iTregs, defined here as CD4^+^ iTregs generated in the presence of naïve CD8^+^ T cells in subsequent experiments. Functional assays demonstrated that these CD4-8 iTregs possess an enhanced capacity to suppress T cell proliferation in vitro with a dose dependent effect (Fig. 1e and f), further supporting their superior regulatory function.

Next, we analyzed phenotypic characteristics in CD8^+^ T cells within the co-culture system. Although the frequency of CD8^+^ T cells was reduced in the co-culture system (Fig. S1e), their absolute numbers showed a trend toward increase, despite not reaching statistical significance. Importantly, these CD8^+^ T cells exhibited minimal production of inflammatory cytokines, including IL-17A, TNF-α, and IFN-γ. In contrast, co-cultured CD8^+^ T cells displayed high CD103 expression and a low percentage of Foxp3^+^ cells (Fig. S1f). Given our previous identification of CD103 as a marker of in vitro–induced CD8 iTregs [30], these findings suggest that CD8^+^ T cells undergo regulatory conversion upon co-culture.

To further investigate the influence of TCR signal strength, we used varying concentrations of anti-CD3/CD28–coated beads to go through graded levels of TCR stimulation. Across all stimulation conditions, CD8^+^ T cells consistently enhanced the frequency of CD39^+^ in iTreg cells (Fig S1 g-h). Moreover, increased CD8^+^ T cell abundance further promoted CD39 expression on CD4 iTregs, a typical dose-dependent effect (Fig S1 i-j). Moreover, as CD8^+^ T cells progressively transitioned from a naïve state (CD62L^+^CD44⁻) to recently activated (CD25^+^CD69^+^), effector (CD62L⁻CD44^+^), and memory-like (CD62L^+^CD44^+^) subsets, their capacity to enhance CD39 expression on CD4 iTregs was progressively diminished (Fig S1 k–l). Together, these data indicate that CD8^+^ T cells promote CD4 iTreg differentiation in a manner dependent on their abundance and activation state.

### CD8^+^ Naïve T cell enhance CD4^+^ iTreg differentiation in vivo

**Fig. 2:**
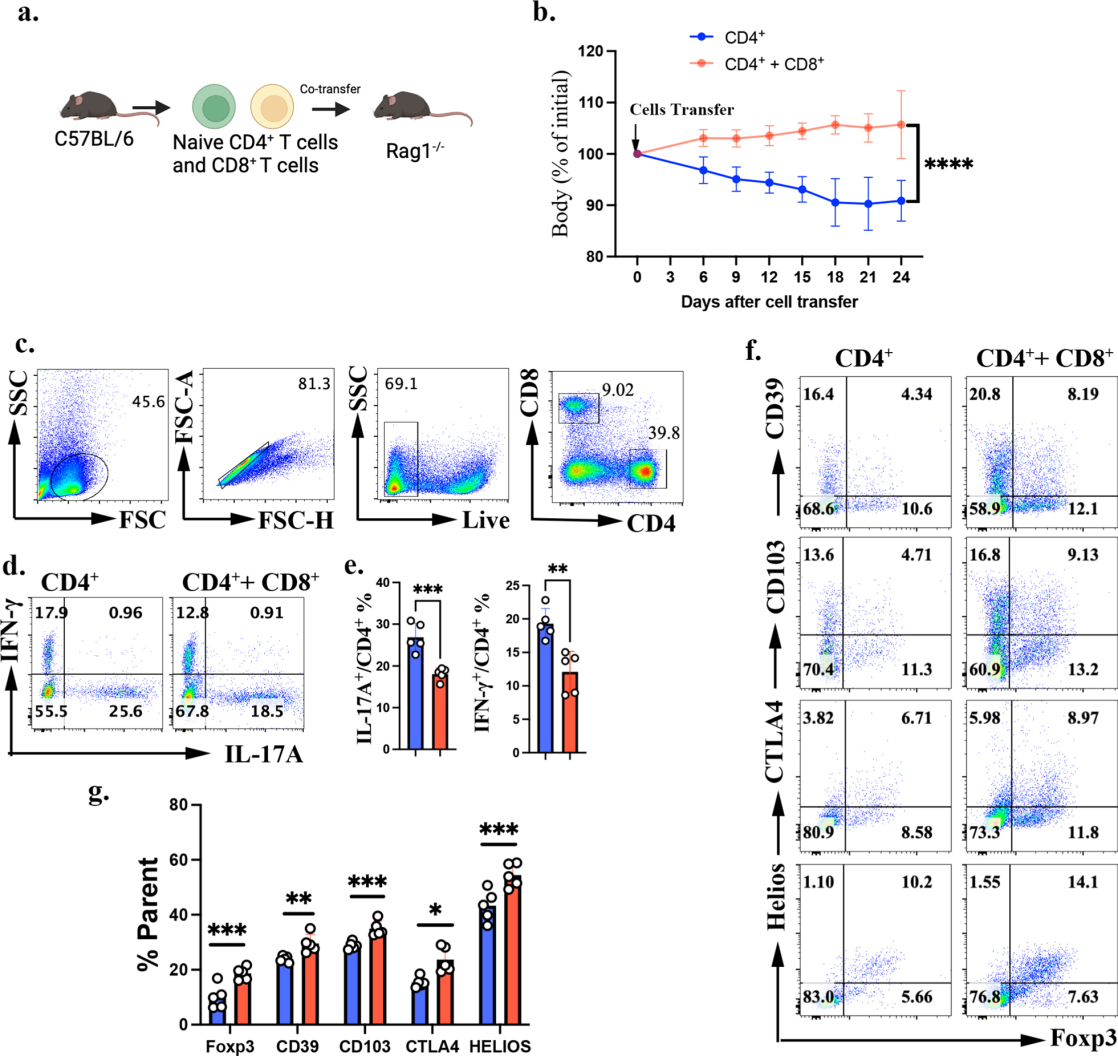
CD8^+^ T cells enhance CD4^+^ iTreg function in vivo. **a** Illustration of the adoptive T cell transfer model of colitis with 0.5 X 10^6^ splenic naïve TCRβ^+^CD4^+^CD62L^+^CD44-Foxp3-GFP- T cells and/or 0.5 X 10^6 TCRβ^+^CD8^+^CD62L^+^CD44- T cells transferred into *Rag1*^-/-^ mice (n = 5 each group); (**b**). Body weight measurements of recipient *Rag1*^-/-^ mice; (**c**). The gating strategy of CD4^+^ T cells; (**d**–**e**). IL-17A^+^ and IFN-γ^+^ percentage in the colon were determined by flow cytometry; (**f**–**g**). Representative flow cytometry data (**f**) and statistic data(**g**) of colon Treg phenotype were shown; Data are representative of three independent experiments. *p* values were calculated by Students’ t test (**e**, **g**). Data are presented as mean ± s.e.m.*p < 0.05, **p < 0.01, ***p < 0.001, ****p < 0.0001,ns, not significant

To further investigate the effects of naïve CD8^+^ T cells on CD4^+^ iTreg induction in vivo, we used the T cell transfer colitis model by adoptively transferring these cell types separately or in combination (Fig. 2a). Our results showed that co-transfer of naïve CD8^+^ T and CD4^+^ T cells significantly alleviated naïve CD4^+^ T cell induced colitis, as evidenced by reduced weight loss (Fig. 2b). Additionally, co-transfer of naïve CD8^+^ T cells markedly suppressed Th1 and Th17 cytokine secretion in CD4^+^ T cells (Fig. 2c-e). Further analysis revealed that co-transfer of naïve CD8^+^ T cells significantly enhanced the generation of CD4^+^ Foxp3^+^ iTreg cells in vivo and increased the frequencies of Helios^+^, CTLA-4^+^, CD39^+^, and CD103^+^ cells within the iTreg population (Fig. 2f-g). Although CD8^+^ T cells did not promote Foxp3 percentage under in vitro conditions, they enhanced Foxp3 induction in vivo, likely driven by additional cytokine and costimulatory signals from the in vivo microenvironment. These findings suggest that naïve CD8^+^ T cells can promote CD4^+^ iTreg differentiation and function in vivo, contributing to immune regulation and colitis attenuation.

To exclude the contribution of cell number differences to disease outcomes, naïve or memory CD8^+^ T cells were co-transferred with equal numbers of naïve CD4^+^ T cells into *Rag1*^⁻/⁻^ mice. Mice receiving naïve CD8^+^ T cells alone maintained significantly higher body weight than those receiving memory CD8^+^ T cells (Fig. S2a), indicating improved disease control. At the endpoint, CD8^+^ T-cell frequencies in the spleen were comparable between two groups, whereas CD8^+^ T-cell frequency in mesenteric lymph nodes (mLN) was higher in the naïve CD8 group. Notably, although the absolute number of CD8^+^ T cells was decreased in the spleen of mice receiving naïve CD8^+^ T cells, only a similar trend—without reaching statistical significance—was observed in the mesenteric lymph nodes (mLN) (Fig. S2b-d). Analysis of co-transferred CD4^+^ T cells revealed that mice receiving memory CD8^+^ T cells exhibited increased Th1 and Th17 differentiation and reduced Treg frequencies, indicating that memory CD8^+^ T cells fail to support CD4^+^ Treg generation and colitis suppression (Fig.S2e-g). CD8^+^ T cells from both groups showed minimal IFN-γ and IL-17A expression; however, TNF-α expression was elevated in memory CD8^+^ T cells (Fig.S2h-i). Immunoregulatory markers IL-10 and CD103 were comparable, whereas CD39 expression significantly reduced in memory CD8^+^ T cells than that in navie CD8^+^ T cells (Fig.S2j-k). Importantly, upon transfer of naïve CD8^+^ T cells alone, naïve and memory CD8^+^ T cells displayed no differences in cell number (Fig. S2d), TNF-α (Fig. S2i), IL-10 (Fig.S2j), or CD39 (Fig.S2k) percentage.Together, these data demonstrate that naïve CD8^+^ T cells suppress colitis indirectly by promoting CD4^+^ iTreg differentiation, rather than through intrinsic immunoregulatory activity.

To further investigate the in vivo interactions between CD8^+^ T cells and CD4^+^ Tregs in human, we analyzed publicly available single-cell transcriptomic data encompassing 13,887 colonic T cells obtained from healthy controls and from ulcerative colitis (UC) patients at different stages, including active disease, paired healthy tissue, and remission. Using Seurat for clustering and UMAP for visualization, we identified multiple T cell subpopulations characterized by distinct marker genes (Fig. S3a-b). The proportions of these subpopulations differed significantly across disease stages, with notably higher frequencies of CD4^+^ T cells and Tregs in the active and paired groups (Fig. S3c). Correspondingly, the number of signaling pathways mediating interactions between CD8^+^ T cells and Tregs was markedly increased (Fig. S3d), suggesting a positive correlation between enhanced Treg expansion and intensified CD8–Treg crosstalk. Subsequent cell–cell communication analysis revealed robust interactions between each CD8^+^ T cell subpopulation and Tregs during the active phase, with the strength of these signaling pathways undergoing dynamic shifts throughout disease progression. Particularly, IL-10 signaling in Tregs was markedly upregulated in the active stage, whereas CD39 signaling was more prominent in the remission stage, implying its potential role in restoring immune homeostasis (Fig. S3f-g). Further investigation showed that Tregs in both the active and paired groups exhibited high expression of signature genes such as CD25, CD103, CD39, and CTLA4(Fig. S3h). Collectively, these findings suggest that interactions between CD8^+^ T cells and CD4^+^ Tregs may be crucial for maintaining the CD4^+^ Treg phenotype.

### CD4-8 iTregs present better function and stability in suppressing naïve CD4^+^ T cell transferred colitis model.

**Fig. 3:**
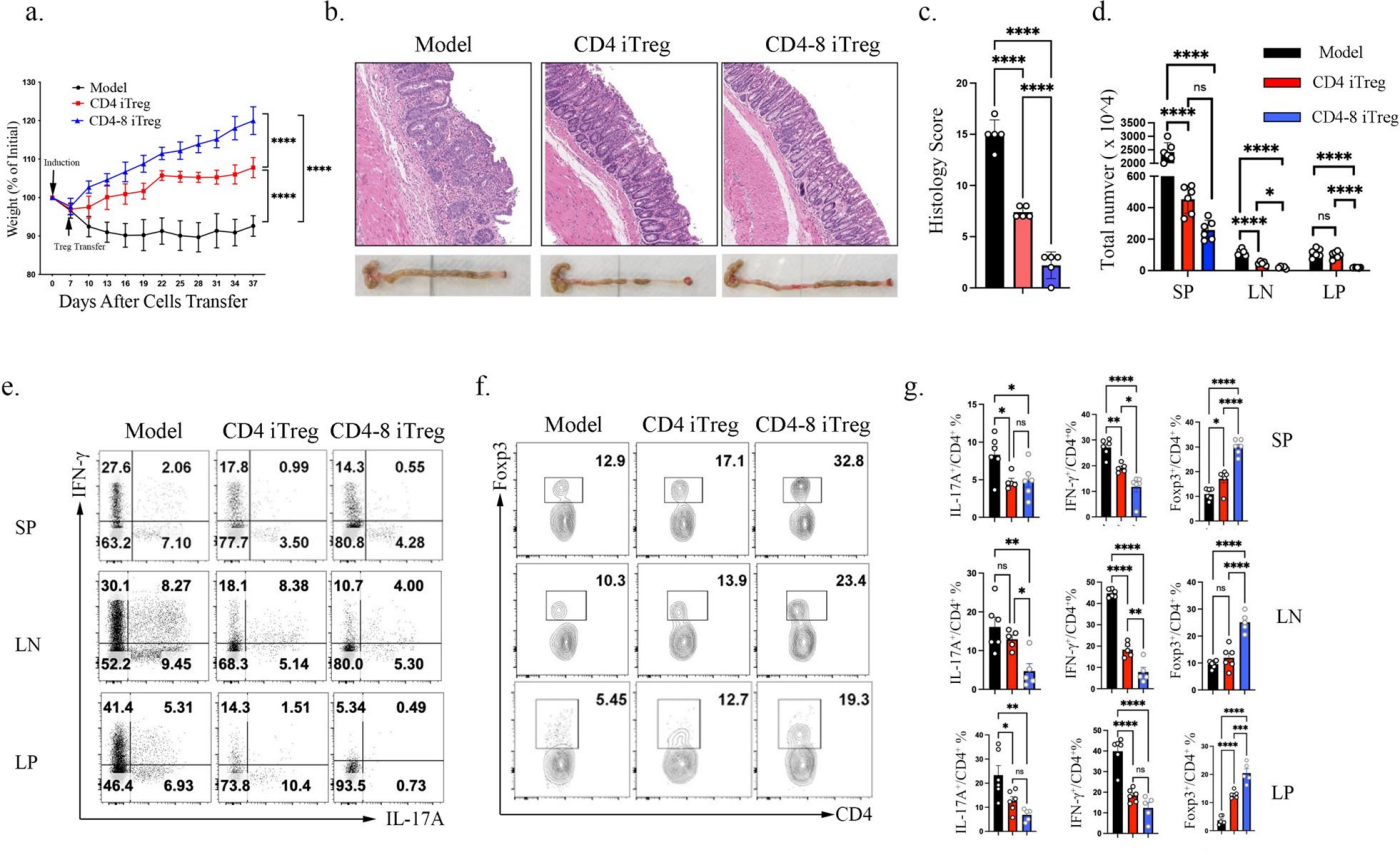
CD4-8 iTregs present enhanced function in suppressing naive CD4^+^ T cell transferred colitis model. CD45.1^+^ naive TCRβ^+^ CD4^+^ T cells were isolated and transferred into *Rag1*^*-*/-^ recipients to induce colitis. Seven days later, CD4^+^Foxp3^+^ iTregs were isolated from in vitro–generated CD45.2^+^ CD4^+^ iTreg cultures, either with or without CD8^+^ T cell priming, and adoptively transferred into colitis mice. **a**. Body weights were recorded every three days after 7 days of model induction; **b**. Gross morphology and HE staining of colons were presented; **c**. Pathological scores of HE staining were shown; **d**. Total live immune cells were counted in spleen, lymph nodes and LP; **e**–**g**. Flow cytometry were used to determined the percentage of IL-17A^+^, IFN-γ^+^ and Foxp3^+^ in CD45.1^+^ CD4^+^ T cells, the represent flow data (**e**,**f**) and the statistical data (**g**) were presented. Data are the combination of two independent experiments, n = 4-6 mice per group, *p* values were calculated by students’ t test (**b, d**) or one-way ANOVA (**f**) followed by Tukey’s multiple comparisons test. Data are presented as mean ± s.e.m.*p < 0.05, **p < 0.01, ***p < 0.001, ****p < 0.0001,ns, not significant.

To assess the functionality and stability of CD4-8 iTregs in vivo, we induced colitis by transferring naïve CD45.1^+^ CD4^+^ T cells into *Rag1*^**⁻/⁻**^ mice via intraperitoneal injection. Naïve CD4^+^ and CD8^+^ T cells from CD45.2 mice were cultured under iTreg-inducing conditions, either separately or in combination. After three days, CD4^+^ Foxp3^+^ iTregs were sorted from both groups and treat colitis mice. Our results showed that both types of iTregs alleviated colitis symptoms. However, compared to CD4^+^ iTregs, CD4-8 iTregs exhibited superior therapeutic efficacy, as evidenced by less reductions in weight loss (Fig. 3a), lower histopathological scores, and significantly decreased colonic swelling (Fig. 3b, c). Additionally, CD4-8 iTreg-treated mice displayed a more pronounced reduction in lymphocyte expansion in the spleen and lymph nodes, along with decreased immune cell infiltration into the colon (Fig. 3d). CD4-8 iTreg-treated colitis mice also showed a significant reduction in Th17 (IL-17A^+^ CD45.1^+^ CD4^+^) cells, whereas CD4^+^ iTregs only significantly suppressed Th17 cells in the spleen and LP. Th1 (IFN-γ^+^ CD45.1^+^ CD4^+^) cells were reduced in the spleen, LN, and LP in both treatment groups, but CD4-8 iTregs exhibited stronger suppression in the spleen and LN, except in the LP. Additionally, CD4-8 iTregs significantly increased Foxp3^+^ CD45.1^+^ CD4^+^ Tregs compared to both model and CD4^+^ iTregs treatment groups, except in the LN (Fig. 3e–g). Collectively, these results indicate that CD4-8 iTregs exhibit superior immunoregulatory capacity in the colitis model, demonstrating enhanced suppression of pro-inflammatory Th1 and Th17 cells while promoting Treg induction.

**Fig. 4:**
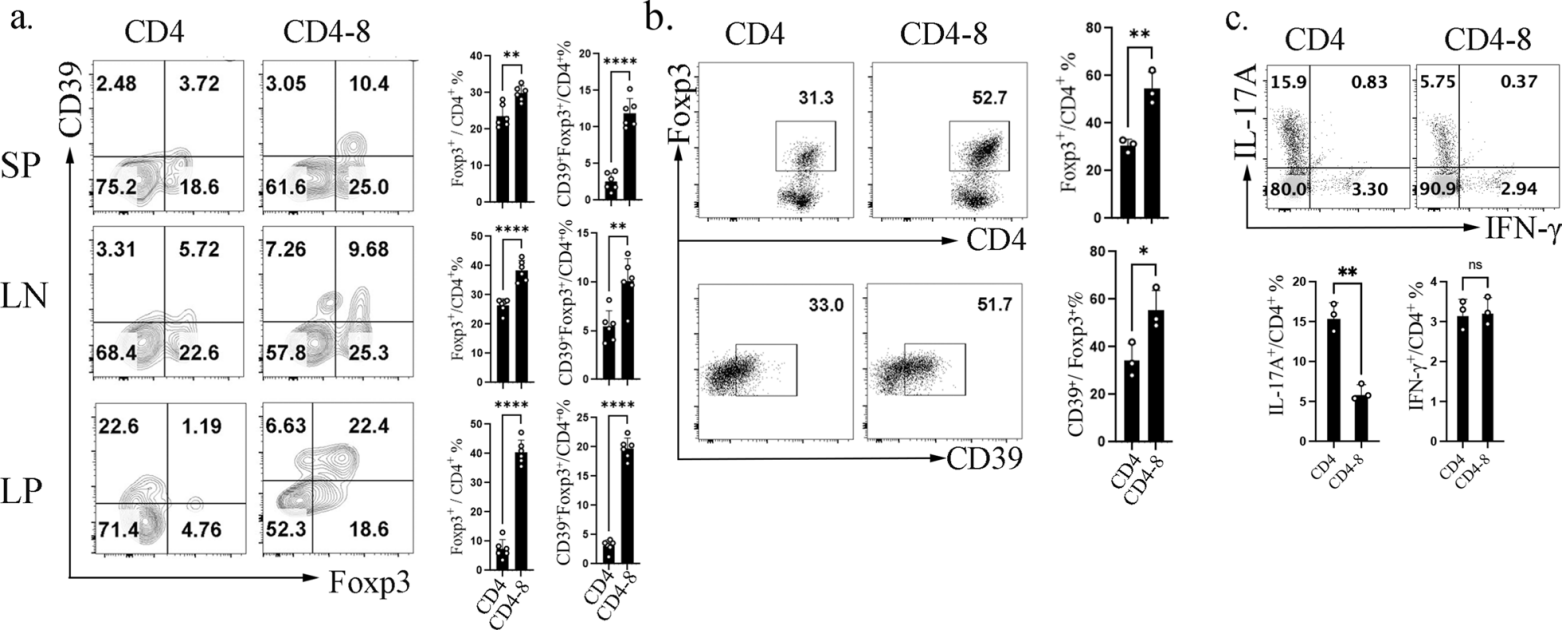
CD4-8 iTregs are more stable in vitro and vivo. **a** CD45.2^+^ CD4 iTreg and CD4-8 iTreg were transferred to colitis mice, the frequency of Foxp3^+^ and CD39^+^ in CD45.2^+^CD4^+^ were determined; **b**–**c** Foxp3^+^ iTregs sorted from CD4-only and CD4–CD8 co-culture systems were restimulated with anti-CD3/CD28 and IL-6 for 5 days in the presence of mitomycin C–treated APCs. CD39 and Foxp3 expression were analyzed in (**b**), whereas inflammatory cytokine production (IL-17A and IFN-γ) was presented in (**c**). Data are representative of three independent experiments, *p* values were calculated by students’ t test, Data are presented as mean ± s.e.m.*p < 0.05, **p < 0.01, ***p < 0.001, ****p < 0.0001; ns, not significant

Treg stability is essential for their immunoregulatory function. While some studies suggest that iTregs are unstable due to high Foxp3 CNS2 methylation, others report their stability under various inflammatory conditions [31, 32]. Our analysis revealed that high-purity naïve T cell-induced CD4^+^ iTregs rapidly lost Foxp3 expression in vivo across the spleen, LN, and LP, with the most pronounced loss in the inflamed LP (CD45.2^+^ CD4^+^ gated). In contrast, CD4-8 iTregs maintained significantly higher Foxp3 percentage and exhibited elevated CD39 proportions (Fig. 4a), a marker linked to Treg stability. To directly assess the stability of the Foxp3 under inflammatory challenge, Foxp3^+^ iTregs were sorted from CD4-only and CD4–CD8 co-culture systems and restimulated with anti-CD3/CD28 and IL-6 in the presence of mitomycin C–treated APCs. iTregs generated in the presence of naïve CD8^+^ T cells maintained significantly higher Foxp3 expression compared with those derived from CD4-only cultures (Fig. 4b). In both groups, CD39^+^ iTregs were preferentially maintained, supporting an association between CD39 expression and increased resistance to inflammatory reprogramming (Fig. 4b). Notably, inflammatory restimulation revealed that CD4–CD8–derived iTregs were less prone to acquire IL-17A expression within the Foxp3^+^ gate, whereas IFN-γ expression was comparable between groups, indicating reduced inflammatory deviation and enhanced lineage stability (Fig. 4c).These results suggest that CD4-8 iTregs exhibit enhanced stability both in vivo and in vitro, underscoring their potential as a more effective therapeutic Treg subset.

### CD8^+^T cell enhanced the proportion of CD39^+^ cells within the CD4-8 iTreg via ROS/TGFβ signaling

**Fig. 5:**
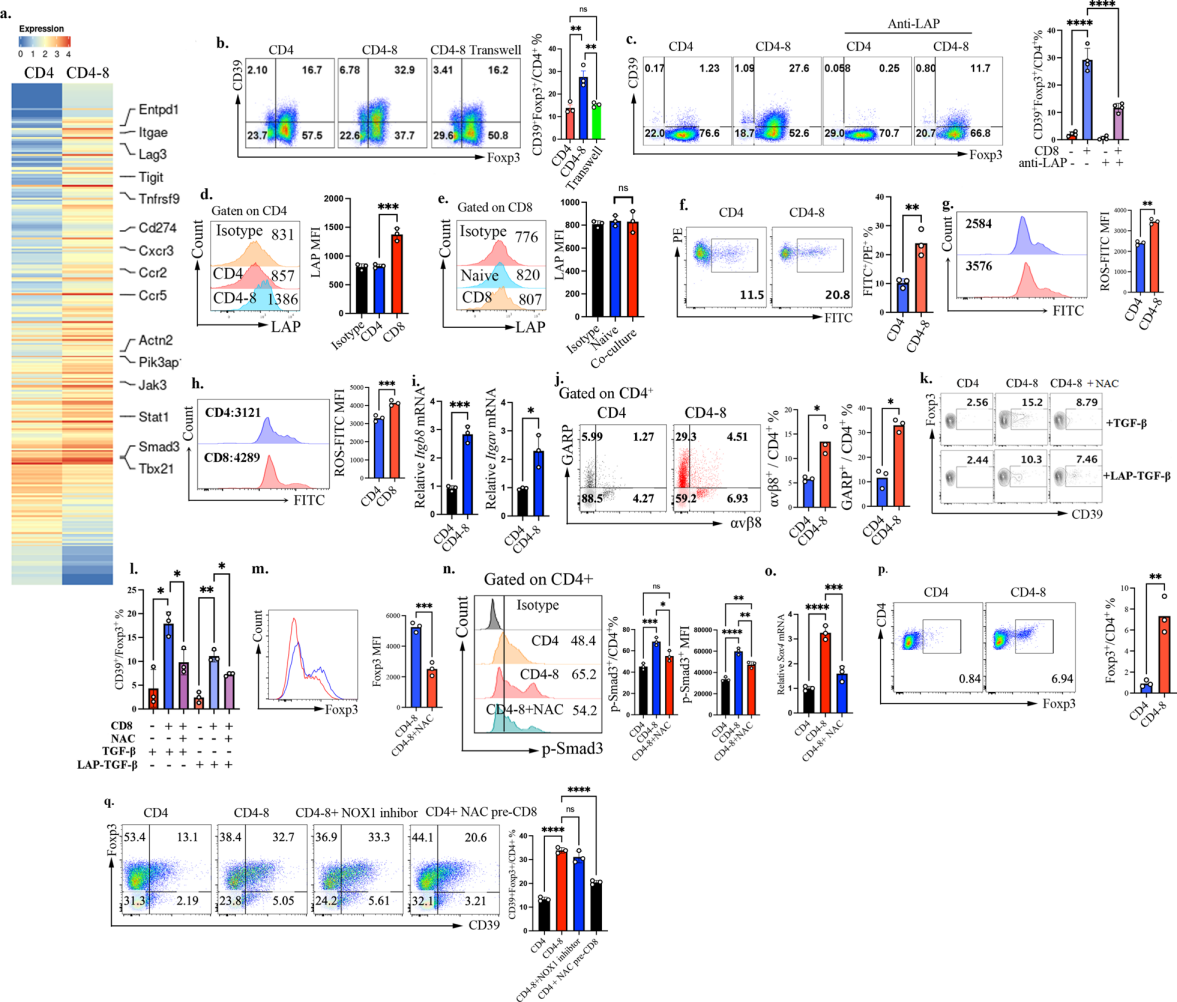
CD8^+^ T cell enhanced the proportion of CD39^+^ cells within the CD4-8 iTreg via ROS/TGFβ signaling*.* **a** CD4^+^ T cells were sorted from CD4 iTreg or CD4-8 iTreg and subjected to RNA sequencing analysis; **b**. A transwell assay were performed to test whether the effect of CD8^+^ T cells is dependent on cell-to-cell contact; **c**. Blocking LAP with a neutralizing antibody significantly reversed the proportion of the CD39 on CD4^+^ iTreg cells by CD8^+^ T cells; **d**. Flow cytometry analysis of LAP expression, quantified as mean fluorescence intensity(MFI), on CD4^+^ T cells from CD4 or CD4-8 groups; **e**. Flow cytometry showing LAP expression, quantified as MFI, on naive CD8^+^ T cells versus activated CD8^+^ T cells from the CD4-8 co-culture system; **f**, **g**. The total ROS production were determined by Bodipy CII(f) and DCFHDA staining(g); **h** Flow cytometric analysis of ROS levels, expressed as MFI, in CD4^+^ iTregs and CD8^+^ T cells in the CD4-8 co-culture system; **i** qRT–PCR analysis of *Itgb8* and *Itgav* expression; **j** Flow cytometric analysis of GARP and αvβ8 expression on CD4^+^ T cells after 48 h of CD4–CD8 co-culture; **k**, **l**.The ROS scavenger NAC significantly reversed the proportion of the CD39 marker on CD4^+^ iTreg cells by CD8^+^ T cells. **m**. CD4^+^Foxp3^+^ Treg cells were isolated from CD4–CD8 co-culture systems with or without NAC treatment. Purified Tregs were subsequently restimulated with anti-CD3/CD28 and IL-6, and Foxp3 expression was analyzed by flow cytometry and quantified as MFI. **n** Expression of p-Smad3 in CD4^+^ T cells from the CD4 and CD4-CD8- groups following iTreg stimulation; **o** qRT–PCR analysis of *Sox4* expression; **p** Flow cytometry showing Foxp3 induction in naive CD4^+^ T cells cultured alone or with naive CD8^+^ T cells without exogenous TGF-β; **q** Flow cytometric analysis of CD39 expression on CD4^+^ iTregs following NOX1 inhibition or NAC pretreatment of CD8^+^ T cells in the co-culture system.Data are representative of three independent experiments, *p* values were calculated by students’ t test (f,g,h,i,j,m and o) or one-way ANOVA (b,c,d,e,l,n and p) followed by Tukey’s multiple comparisons test, Data are presented as mean ± s.e.m.*p <0.05, **p <0.01, ***p <0.001, ****p <0.0001, ns, not significant.

To further elucidate the mechanisms by which CD8^+^ T cells regulate iTreg differentiation and function, we performed RNA sequencing on induced CD4^+^ T cells isolated from these two groups. A total of 277 differentially expressed genes (|Fold Change|> 2) were identified. Among these were genes associated with Treg immunosuppressive function**,** including CD103, CD39, LAG3, TIGIT, 4-1BB, and PD-L1**,** were upregulated. Additionally, genes involved in cytoskeletal organization and migration**,** such as CXCR3, CCR2, and ACTN2, were also increased (Fig. 5a and Table S1). These findings suggest that CD8^+^ T cells may enhance iTreg function by promoting the expression of immunoregulatory molecules and facilitating cellular migration. We also found that a substantial proportion of differentially expressed genes were associated with cytokine signaling, with a significant upregulation of JAK3 and STAT1 expression. Given the crucial role of CD39 in iTreg function and stability, we selected CD39 as an indicator molecule to assess the regulatory impact of CD8^+^ T cells on CD4 iTregs. Using a Transwell co-culture system, we found that CD8^+^ T cell-induced upregulation of CD39 in iTregs was dependent on direct cell–cell contact (Fig. 5b).

Considering that both CD39 and CD103 are regulated by the TGF-β signaling pathway, and that the presence of CD8^+^ T cells leads to an increased expression of Smad3 (Fig. 5a), we hypothesize that CD8^+^ T cells may enhance TGF-β signaling, thereby promoting CD39 expression in iTregs. Given that TGF-β is present in the system, we further propose that CD8^+^ T cells may facilitate endogenous TGF-β activation by promoting LAP cleavage, thereby amplifying TGF-β signaling. To validate this hypothesis, we examined the effect of blocking endogenous TGF-β activation. Notably, treatment with anti-LAP antibody significantly reversed the CD8^+^ T cell-induced increase for the frequency of CD39^+^ cells within CD4^+^ iTregs (Fig. 5c**),** supporting that CD8^+^ T cells enhance iTreg function by promoting LAP cleavage and activating endogenous TGF-β signaling. We next sought to determine the cellular source of endogenous LAP. Our analyses revealed that CD4^+^ T cells did not exhibit detectable LAP expression, as measured by mean fluorescence intensity(MFI), during iTreg differentiation. However, in the presence of CD8^+^ T cells, LAP expression(MFI) on CD4^+^ T cells was markedly increased (Fig. 5d). In contrast, LAP expression(MFI) on CD8^+^ T cells was not significantly altered compared with naïve T cells (Fig. 5e). These data indicate that endogenous LAP is primarily derived from CD4^+^ T cells themselves.

Moreover, we found that CD8^+^ T cells also led to a marked increase in reactive oxygen species (ROS) levels in iTregs (Fig. 5f, g). Further analysis revealed that, within the co-culture system, CD8^+^ T cells themselves exhibited significantly higher levels of ROS(quantified as MFI), indicating that ROS is derived not only from CD4^+^ T cells but also from CD8^+^ T cells (Fig. 5h). Several molecules have been implicated in TGF-β activation in immune cells, including integrin αvβ8 (encoded by the Itgav and Itgb8 subunits) and GARP. Consistent with this, we observed that the presence of CD8^+^ T cells significantly increased the mRNA and protien expression of *Itgav* and *Itgb8* in CD4^+^ T cells under co-culture conditions (Fig. 5i, j). In addition, GARP, which is known to anchor LAP on the cell surface, was also upregulated, indicating that both the αvβ8 integrin complex and GARP-mediated LAP presentation are enhanced in the presence of CD8^+^ T cells (Fig. 5j).

Given that ROS has been implicated in various aspects of TGF-β signaling, including the regulation of LAP cleavage and Smad3 activation, we hypothesized that ROS might serve as an intermediary in CD8^+^ T cell-mediated TGF-β activation. To test this, we treated CD4 iTregs with NAC (a ROS inhibitor) and observed a significant reversal of CD8^+^ T cell induced CD39 upregulation (Fig. 5k,l). We further confirmed that iTregs generated in the CD4–CD8 co-culture system exhibited enhanced Foxp3 maintenance upon anti-CD3/CD28 plus IL-6 restimulation, whereas NAC treatment during co-culture diminished this Foxp3 stability**,** reflected by reduced Foxp3 MFI upon restimulation (Fig. 5m).Moreover, the presence of CD8^+^ T cells markedly increased Smad3 phosphorylation in iTregs, an effect that was effectively abrogated by NAC treatment (Fig. 5n). Sox4 has been reported to play a critical role in TGF-β–induced CD39 expression [33]. Consistent with this notion, we observed a significant increase in Sox4 expression in iTregs cultured in the presence of CD8^+^ T cells, which was also reversed upon NAC treatment (Fig. 5o). Next, we tested whether naïve CD4^+^ T cells co-cultured with naïve CD8^+^ T cells could generate iTregs in the absence of exogenous TGF-β. Foxp3 induction was minimal under these conditions, indicating that CD8^+^ T cells do not initiate iTreg differentiation but instead amplify TGF-β signaling once it is engaged (Fig. 5p). Finally, we found that inhibition of NOX1 failed to reverse CD39 expression in CD4^+^ iTregs, whereas pretreatment of CD8^+^ T cells with NAC markedly attenuated CD39 upregulation in CD4^+^ iTregs (Fig. 5q).Together, these findings suggest that CD8^+^ T cells promote iTreg function not only by facilitating LAP cleavage but also by increasing ROS levels, which in turn amplify TGF-β signaling and downstream Smad3–Sox4–CD39 transcriptional programs.

### The expression of IRF4 in CD8 is essential for its function in iTreg generation.

**Fig. 6:**
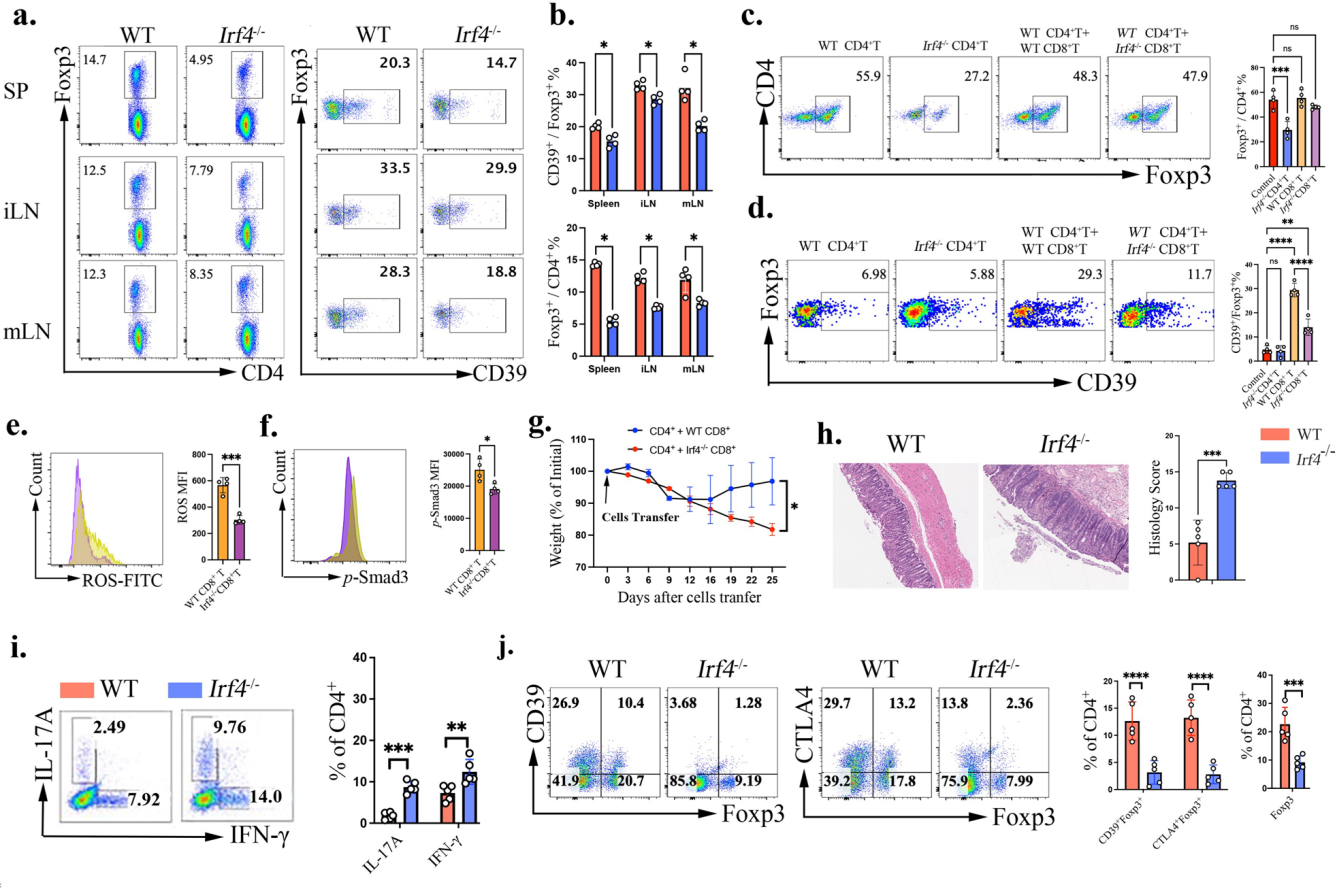
The expression of IRF4 in CD8 is essential for its function in CD4 iTreg generation. **a**, **b**. Flow cytometry was performed to analyze the frequency of Tregs and the proportion of CD39 in Tregs from the spleen, inguinal lymph nodes, and mesenteric lymph nodes of WT and *Irf4*^−/−^ mice. Representative flow cytometry plots are shown in (**a**), with quantification presented in (**b**); **c**, **d**. WT naive CD4^+^ T cells were co-cultured at a 1:1 ratio with naive CD8^+^ T cells from either WT or *Irf4*^*⁻/⁻*^ mice under iTreg-polarizing conditions, and the percentage of Foxp3 (**c**) and CD39 (**d**) was assessed by flow cytometry, the representative flow data (left panel) and the statistical data(right panel) were presented; **e**, **f**. Flow cytometric analysis of intracellular ROS (**e**) and p-Smad3 (**f**), quantified as MFI, in CD4^+^ T cells under the indicated co-culture conditions. Representative plots and quantification within the Foxp3^+^ gate are shown. **g**–**j** A total of 0.5 × 10⁶ splenic naive TCRβ^+^ CD4^+^CD62L^+^CD44-Foxp3-GFP⁻ T cells were co-transferred into *Rag1⁻/⁻* mice along with 0.5 × 10⁶ TCRβ^+^ CD8^+^CD62L^+^CD44- T cells isolated from either WT or *Irf4*^*⁻/⁻*^ mice. Body weight was monitored every three days following cell transfer (**g**) At the study endpoint, H&E staining of colonic tissue was performed (**h**); Flow cytometry was used to evaluate the proportion of IL-17A, IFN-γ(i). Flow cytometry was used to evaluate the proportion of Foxp3, CTLA-4, and CD39 (**j**). Data are representative of three independent experiments, *p* values were calculated by students’ t test (**e**, **f**, **h**, **i**, **j**) or oneway ANOVA (**c**, **d**, **g**). Data are presented as mean ± s.e.m.*p < 0.05, **p < 0.01, ***p < 0.001, ****p < 0.0001, ns, not significant.

To understand the underlying mechanism, we sought to determine the key factors within CD8^+^ T cells that are essential for this regulatory process. IRF4 (Interferon Regulatory Factor 4) has been previously identified as a critical transcription factor for Treg differentiation and function, playing a key role in maintaining their stability and immunosuppressive activity [34]. Additionally, IRF4 regulates CD8^+^ T cell activation, differentiation, and metabolic reprogramming**,** particularly in response to TCR stimulation and inflammatory signals [35]. This raises the question of whether IRF4 is involved in the immunoregulatory function of CD8^+^ T cells in promoting CD4 iTregs. Consistent with previous reports, we observed that *Irf4*^−/−^ mice exhibited a reduced Treg proportion in the spleen, inguinal lymph nodes, and mesenteric lymph nodes (Fig. 6a, b). Interestingly, CD39 proportion was also significantly reduced (Fig. 6a, b), suggesting that IRF4 influences not only Treg homeostasis but also the expression of key immunoregulatory molecules. However, beyond its direct impact on CD4^+^ T cells, we investigated whether IRF4 could also influence Treg phenotype through its effects on CD8^+^ T cells. In vitro*,* IRF4 deficiency in CD4^+^ T cells reduced iTreg differentiation, but notably, CD39 percentage was not significantly affected (Fig. 6c, d). However, when IRF4 was specifically deleted in CD8^+^ T cells, their ability to increase the frequency of CD39^+^ cells within the CD4^+^ iTreg cells was lost (Fig. 6c, d). We next examined whether IRF4-deficient CD8^+^ T cells regulate CD39 expression on iTregs through the ROS–TGF-β signaling axis. Compared with WT CD8^+^ T cells, IRF4-deficient CD8^+^ T cells exhibited significantly reduced intracellular ROS levels, quantified as MFI, and diminished *p*-Smad3 signaling, also measured as MFI, in the co-culture system(Fig. 6e, f). These findings indicate that IRF4 expression in CD8^+^ T cells is required to sustain ROS–TGF-β signaling, thereby promoting CD39 expression and stabilizing the iTreg program.

Then, we co-transferred CD4^+^ naive T cells with either WT or *Irf4*^−/−^CD8^+^ T cells (1:1 ratio) into *Rag1*^−/−^ mice. Mice receiving *Irf4*^−/−^CD8^+^ T cells exhibited significantly greater weight loss and higher disease severity scores than those receiving WT CD8^+^ T cells(Fig. 6g-h). Flow cytometry analysis revealed that the proportion of pro-inflammatory Th1 and Th17 cells was markedly increased in mice with *Irf4*^−/−^ CD8^+^ T cells, while Tregs were significantly reduced(Fig. 6i–j)**.** Furthermore, the frequencies of CD39^+^ and CTLA-4^+^ cells within the iTreg population were also significantly reduced in the *Irf4*^−/−^CD8^+^ T cell group (Fig. 6j). These findings collectively demonstrate that IRF4 is crucial for CD8^+^ T cell-mediated induction of iTregs. The loss of IRF4 disrupts CD8^+^ T cell-dependent immune regulation**,** leading to increased inflammation and impaired Treg function**.** This highlights a previously unrecognized role of IRF4 in CD8^+^ T cells**,** where it facilitates the induction of CD39^+^ iTregs**.**

### CD8^+^ T Cells enhance the therapeutic potential of CD4^+^ iTregs in autoimmune arthritis

**Fig. 7:**
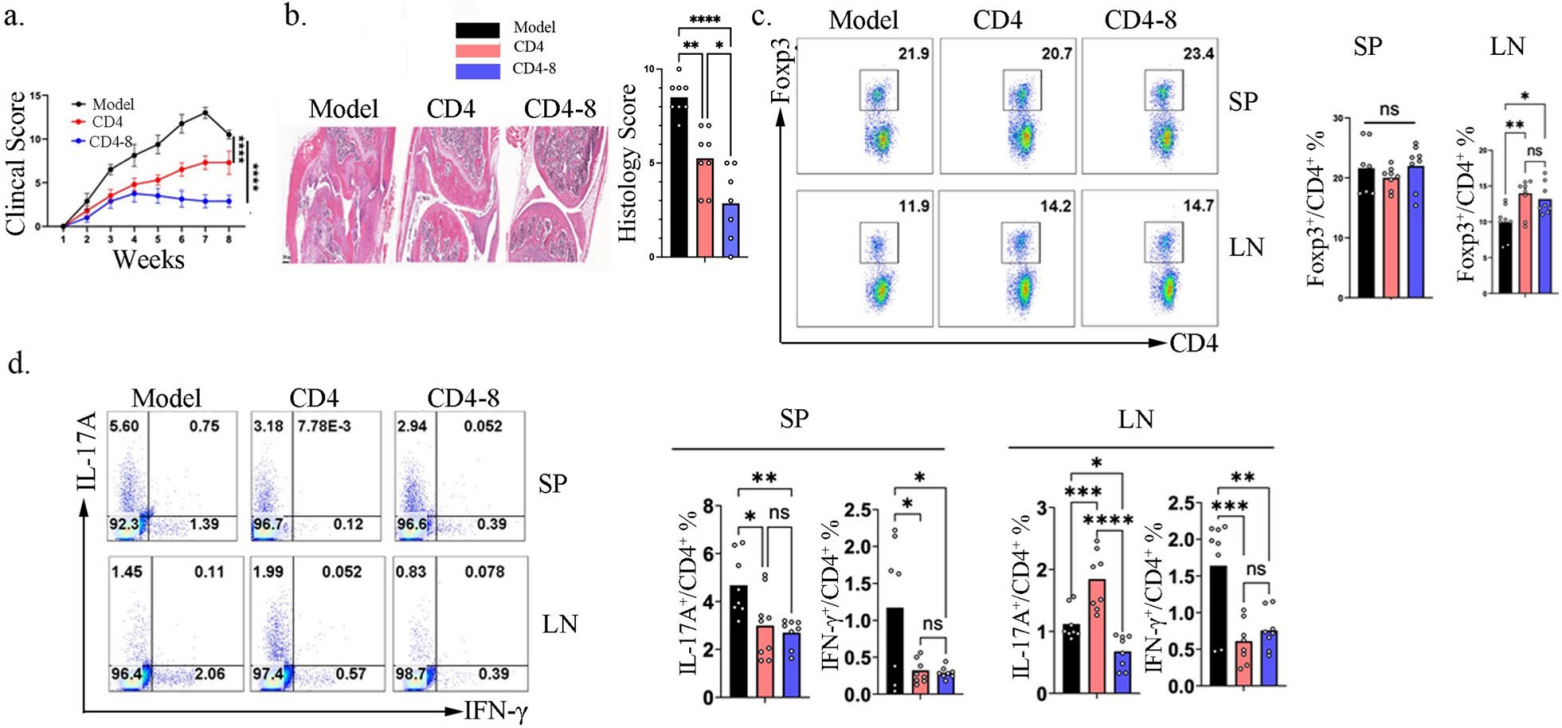
CD8^+^ T primed CD4^+^ iTreg cell present better function in suppressing collagen induced arthritis. CD4^+^ iTreg and CD8^+^ Primed CD4 ^+^ iTreg were isolated and used to treat established CIA(about 30 days after immunization), the mice were euthanized after 30 days of iTreg transfer. (**a**). Clinical score were recorded every three days after CII/CFA immunization; (**b**). HE staining of joint and pathological scores were presented; (**c**, **d**). Flow cytometry were used to determined the percentage of Foxp3^+^ (**c**), IL-17A^+^ and IFN-γ^+^ (**d**) in CD4^+^ T cells. Data are representative of three independent experiments, n = 8 mice per group, *p* values were calculated by two-way ANOVA (**a**) or one-way ANOVA (**b**, **c**, **d**) followed by Tukey’s multiple comparisons test. Data are presented as mean ± s.e.m. *p < 0.05, **p < 0.01, ***p < 0.001, ****p < 0.0001,ns, not significant

To further explore the clinical relevance of CD8^+^ T cells in enhancing the suppressive capacity and stability of CD4^+^ Tregs**,** we investigated whether CD4-8 iTregs could be effective in autoimmune arthritis**.** We utilized the collagen-induced arthritis (CIA) model, as previously described. CD4-8 iTregs or conventional iTregs were intravenously transferred into CIA mice. Fifty-six days post-transfer, the mice were sacrificed, and inflammatory immune cell populations were analyzed by flow cytometry. Consistent with our findings in the colitis model**,** CD4-8 iTregs exhibited therapeutic effects in CIA mice, as evidenced by a reduced clinical score and histological score of arthritis (Fig. 7a, b). Accordingly, Treg populations were maintained in CIA mice receiving CD4-8 iTregs (Fig. 7c). Notably, the proportions of Th1 and Th17 cells were significantly decreased in the spleen and lymph nodes of CIA mice treated with CD4-8 iTregs (Fig. 7d). Collectively, these findings highlight the therapeutic potential of CD4-8 iTregs in autoimmune arthritis**,** further underscoring the role of CD8^+^ T cells in enhancing Treg suppressive function and stability**.**

## Discussion

CD4^+^ and CD8^+^ T cells coordinate to maintain immune homeostasis and execute effective immune functions. It has been reported that CD4^+^ T cells provide critical help for CD8^+^ T cell activation, proliferation, and functional maintenance through mechanisms such as licensing antigen-presenting cells (APCs) [36, 37], secreting cytokines (e.g., IL-2) [38], and promoting memory formation [39]. Moreover, the regulatory effects of CD8^+^ T cells on CD4^+^ T cells are generally considered to be indirect, primarily through the clearance of infected cells to reduce antigen load, or via the secretion of cytokines such as IFN-γ that influence CD4^+^ T cell differentiation [40]. However, the potential role of CD8^+^ T cells in regulating the differentiation, function, and stability of Tregs remains unexplored.

Here, we provide novel insights into the role of CD8^+^ T cells in enhancing the function, and stability of CD4^+^ iTregs. Our findings reveal that CD8^+^ T cells significantly increase the frequencies of CD39^+^, Helios^+^, CTLA-4^+^, and CD103^+^ subsets within iTreg population. Notably, CD8^+^ T cells did not alter the frequency of Foxp3^+^ cells but markedly increasing their absolute numbers. These results suggest that CD8^+^ T cells promote the functional specialization of iTregs, rendering them more potent in immune regulation. Importantly, this effect is not observed with other immune cell subsets, emphasizing the unique role of CD8^+^ T cells in modulating iTreg biology.

The in vivo relevance of these findings was validated in a T cell transfer colitis model, where the co-transfer of CD8^+^ naive T cells significantly alleviated colitis induced by naive CD4^+^ T cells. Mechanistically, CD8^+^ T cells promoted iTreg induction and increased the frequencies of key functional marker of iTreg subsets, thereby suppressing pathogenic Th1 and Th17 responses. These data suggest that CD8^+^ T cells contribute to immune homeostasis by fostering a more suppressive iTreg population which it has never been recognized previously. However, recent studies have also reported that co-transfer of CD8^+^ T cells with a small number of CD4^+^ T cells, which alone are insufficient to induce colitis, can enhance the cytotoxic activity of CD8^+^ T cells and exacerbate intestinal inflammation [41]. This finding raises the possibility that different ratios or activation states of CD4^+^ and CD8^+^ T cells may have a distinct and/or even opposite effect on the development and progression of colitis [41]. Moreover, our data found that when CD4^+^ iTregs were primed with CD8^+^ T cells prior to adoptive transfer, they exhibited superior therapeutic efficacy in mitigating colitis severity, as evidenced by reduced histopathological damage, decreased inflammatory cytokine secretion, and enhanced Treg stability.

Our data show that naïve CD8^+^ T cells, but not CD44^+^ memory CD8^+^ T cells, promote CD4^+^ iTreg differentiation. Several immunological features likely underlie this distinction. First, naïve CD8^+^ T cells have a quiescent basal state with low tonic signaling, which may allow them to provide balanced costimulation without delivering excessive inflammatory cues. Second, naïve CD8^+^ T cells produce minimal effector cytokines, thereby avoiding pathways known to antagonize Foxp3 induction. Third, naïve cells rely primarily on oxidative phosphorylation rather than glycolytic effector metabolism, creating a microenvironment more favorable for TGF-β–driven iTreg programming. Finally, the early-activation transcriptional landscape of naïve CD8^+^ T cells—particularly their IRF4-dependent early activation signature—may more effectively support iTreg stability, whereas memory CD8^+^ T cells rapidly upregulate effector pathways that counteract iTreg differentiation. These mechanistic differences likely explain why naïve but not memory CD8^+^ T cells promote iTreg induction in our system.

A crucial factor influencing iTreg stability and function is the expression of CD39, a molecule associated with Treg suppressive capacity and resistance to inflammatory reprogramming [22]. We here observed that CD8^+^ T cells increased CD39^+^ subset in iTregs via a ROS/TGF-β signaling axis. Interestingly, recent reports have shown that CD8^+^ T cells enriched within tumor microenvironments can promote the generation of T-bet^+^ Tregs, a subset characterized by high CD39 expression [42], which further supports our findings. Mechanistically, CD8^+^ T cells promoted reactive oxygen species (ROS) generation and latency-associated peptide (LAP) cleavage, leading to enhanced TGF-β activation. Inhibition of either ROS or TGF-β signaling significantly abrogated the CD8^+^ T cell-induced upregulation of CD39 proportion, underscoring the importance of this pathway in iTreg functional enhancement. These findings strongly suggest that CD8^+^ naive T cells orchestrate a supportive microenvironment for iTreg stability and function by modulating intracellular signaling cascades.

Our results further revealed that IRF4 modulates CD39^+^ subset in CD4^+^ Tregs, which has never been shown previously. Thus, the ROS/TGF-β pathway presents a novel mechanism that CD8^+^ T cells rheostat CD4^+^ Treg function and stability. The transcription factor IRF4 is essential for robust CD8^+^ T cell responses [35, 43]. Also, IRF4 can maintain Treg function by regulating Foxp3 [35]. However, whether IRF4 can modulate the cell–cell interaction between CD8^+^ T cell and iTregs remain unclear. Here, we revealed that IRF4-deficient CD8^+^ T cells failed to increase CD39^+^ iTreg subset within CD4+ iTregs and were unable to provide effective immune regulation in the colitis model. Moreover, co-transfer of IRF4-deficient CD8^+^ T cells exacerbated disease severity, with increased Th1 and Th17 responses and reduced Foxp3^+^ Treg populations. These findings indicate that IRF4 is indispensable for CD8^+^ T cell-mediated modulation of iTregs, likely through its role in shaping the metabolic and transcriptional landscape of CD8^+^ T cells.

Beyond colitis, we also demonstrated the translational potential of CD8^+^ T cell-primed CD4^+^ iTregs in an autoimmune arthritis model. Similar to their effects in colitis, CD8^+^ T cell-primed iTregs displayed superior therapeutic efficacy in reducing arthritis severity, maintaining Treg populations, and suppressing inflammatory Th1 and Th17 cells. Indeed, these T effector cells mainly involve in arthritis pathogenesis [44, 45]. These results provide strong evidence that CD8+ T cells enhance the therapeutic potential of iTregs across multiple inflammatory disease models, suggesting their broader applicati-on in immune modulation therapies.

## Conclusions

Our study identify a critical and previously unappreciated role of CD8^+^ T cells in promoting the differentiation, stability, and suppressive function of CD39^+^ induced Tregs through a ROS/TGF-β–IRF4 signaling axis. By reinforcing iTreg-mediated control of Th1 and Th17 responses, CD8^+^ T cells significantly ameliorated autoimmune inflammation in both colitis and arthritis models. These findings highlight an important layer of immune regulation between CD8^+^ T cells and Tregs, and provide a mechanistic rationale for harnessing CD8^+^ T cell–primed iTregs as a novel therapeutic approach to enhance Treg-based interventions in autoimmune diseases.

### Material and methods

#### Mice

All mice used in this study, including CD45.1, Foxp3-GFP reporter, *Irf4*^−/−^ and *Rag1*^−/−^ mice, were between 6 and 14 weeks old at the start of the experiments. *Irf4*^−/−^ and *Rag1*^−/−^ mice were purchased from GemPharmatech Co., Ltd. The mice were bred and housed under specific pathogen-free (SPF) conditions at Shanghai Jiao Tong University. Age- and sex-matched mice were used for all experiments, and both male and female mice were analyzed. For consistency, paired or grouped mice used in each experiment were co-housed. All animal procedures were conducted in accordance with the Institutional Animal Care and Use Committee (IACUC) guidelines and the regulations of the animal facility (IACUC # ACE-003–2025).

#### Flow cytometry and antibodies

Flow cytometry staining was performed following standard protocols. Briefly, single-cell suspensions were prepared from harvested tissues by mechanical dissociation and filtration through a 70 μm cell strainer. Red blood cells were lysed using Red Blood Cell Lysing Buffer Hybri-Max (Sigma-Aldrich) where necessary. Cells were washed with PBS containing 2% fetal bovine serum (FBS) and resuspended at an appropriate concentration. For surface staining, cells were incubated with fluorochrome-conjugated antibodies specific to target markers at 4 °C for 30 min in the dark. After staining, cells were washed twice with FACS buffer (PBS + 2% FBS) and resuspended in 200 μL of the same buffer for acquisition. For intracellular staining, cells were fixed and permeabilized using the Fixation/Permeabilization Kit (eBioscience) before incubation with antibodies against intracellular markers such as Foxp3 or cytokines. The following fluorochrome-conjugated antibodies were used for flow cytometry analysis: CD103 (clone 2E7, APC), CD4 (clone GK1.5, APC–Cy7), CD8a (clone 53–6.7, PerCP–Cy5.5), CD25 (clone PC61, BB515), CD62L (clone MEL-14, PE), and CD44 (clone IM7, APC) (BioLegend, San Diego, CA, USA), as well as Helios (clone 22F6, FITC), CD39 (clone Duha59, PE), CTLA-4 (clone UC10-4B9, PE–Cyanine7), and Foxp3 (clone FJK-16 s, PE–Cyanine7) (Thermo Fisher Scientific, Waltham, MA, USA). Antibodies were used at final dilutions of 1:200 for surface markers and 1:100 for intracellular markers unless otherwise indicated. Surface staining was performed for 20 min at 4 °C in PBS supplemented with 2% FBS. For intracellular staining of Foxp3, Helios, and CTLA-4, cells were fixed and permeabilized using a Foxp3/Transcription Factor Staining Buffer Set according to the manufacturer’s instructions (Thermo Fisher Scientific).Stained samples were analyzed on a BD LSRFortessa or BD FACSAria III flow cytometer, and data were processed using FlowJo software V10.

#### CIA model

Collagen-induced arthritis (CIA) was established in DBA/1 mice following a standard immunization protocol [46]. Briefly, bovine type II collagen (CII) was dissolved in 0.05 M acetic acid (2–4 mg/mL) and emulsified with an equal volume of complete Freund’s adjuvant (CFA) using a homogenizer. On Day 0, 100 μL of the CII-CFA emulsion was injected subcutaneously at the base of the tail. On Day 21, a booster injection of 100 μL of CII emulsified in incomplete Freund’s adjuvant (IFA) was administered at the same site. Mice were monitored every 2–3 days for signs of arthritis, including paw swelling and redness, and disease severity was scored using a standard arthritis scoring system. CIA severity was evaluated using a 0–4 clinical scoring system based on erythema and swelling. Scores were defined as follows: 0, no signs of inflammation; 1, erythema and mild swelling limited to the tarsals/ankle; 2, mild swelling extending from the ankle to the tarsals; 3, erythema with moderate swelling reaching the metatarsal joints; 4, severe swelling involving the ankle, foot, and digits or limb ankylosis. All assessments were performed in a blinded manner. Body weight and general health were also assessed. At the experimental endpoint (typically Day 35–42, or when arthritis reached a severe stage), mice were euthanized, and spleen, lymph nodes, and joint tissues were collected for further analysis. Joints were fixed in 4% paraformaldehyde, decalcified, embedded in paraffin, and stained with hematoxylin and eosin (H&E) for histopathological assessment.

#### Colitis model

Spleens from donor mice were harvested, mechanically dissociated in complete RPMI medium (Gibco) (RPMI 1640 supplemented with 10% FBS) and filtered through 70 μm cell strainers. Red blood cells were lysed using Red Blood Cell Lysing Buffer (Sigma-Aldrich). T cells were enriched via the nylon wool column method and subsequently stained with LIVE/DEAD Fixable Dead Cell Stain (BD), anti-mouse TCRβ, CD4, CD62L, CD44 and CD8 antibodies (Biolegend). Live naïve CD4^+^(TCRβ^+^CD4^+^^+^ CD44^−^ Foxp3-GFP^−^) and naïve CD8^+^ (TCRβ^+^CD8^+^CD62L^+^ CD44^−^) T cell populations were sorted using a BD FACS Aria III cell sorter. For the naïve CD4^+^ T cell transfer model, 5 × 105 naive CD4^+^ T cells were intraperitoneally injected into *Rag1*^⁻/⁻^ recipient mice. In the co-transfer model, an equal number (5 × 105) of naïve CD4^+^ and CD8^+^ T cells were co-injected into *Rag1*^⁻/⁻^ mice. Mice were monitored daily for clinical symptoms, including hunching of the back and diarrhea. Body weight was recorded every other day, and mice were euthanized if they exhibited > 15% weight loss from baseline. At the end of the experiment, spleen, mesenteric lymph nodes (mLNs) and distal colons were harvested, with the middle section used for histological analysis. Colons were fixed in zinc formalin for 48 h, followed by H&E staining. The degree of inflammation in the lamina propria (score 0–3), goblet cell loss (score 0–2), crypt architectural abnormalities (score 0–3), presence of crypt abscesses (score 0–1), mucosal erosion and ulceration (score 0–1), submucosal extension to transmural involvement (score 0–3), and neutrophil infiltration quantified by counting neutrophils at × 40 magnification (score 0–4) were assessed. Scores for all seven parameters were summed to generate a total histopathological score with a maximum possible score of 17. All histological assessments were conducted in a blinded and randomized manner.

### Cell purification and differentiation in vitro

Naïve CD4^+^ T cells (TCRβ^+^CD4^+^CD62L^+^ CD44^−^CD25⁻) were isolated from mouse spleen and lymph nodes and cultured in RPMI 1640 medium supplemented with 10% fetal bovine serum (FBS), 1% penicillin/streptomycin, 2 mM L-glutamine, 1 mM sodium pyruvate, and 50 μM β-mercaptoethanol. For iTreg differentiation, cells were stimulated with anti-CD3/28 beads (1:5 to cell) in the presence of IL-2 (50 U/mL) and TGF-β1 (2 ng/mL). Cells were incubated at 37 °C with 5% CO₂ for 3 days, after which they were collected, washed, and stained for surface markers (CD4, CD25) and intracellular Foxp3 using the Foxp3 Staining Kit (eBioscience). iTreg differentiation was assessed by flow cytometry, and the proportion of CD4^+^CD25^+^Foxp3^+^ cells was quantified.

#### CFSE proliferation assay

A carboxyfluorescein succinimidyl ester (CFSE) proliferation assay was performed to evaluate the suppressive function of CD4^+^Foxp3^+^ iTreg cells generated in the presence or absence of CD8^+^ T cells. Briefly, sorted CD3^+^ T cells were resuspended in PBS (without FBS) at a concentration of 1 × 10⁷ cells/mL. Cells were stained with CFSE (5 μM; Invitrogen) for 10 min at 37 °C in the dark. After washing twice with complete medium, labeled cells were co-cultured with Mitomycin C-treated APCs in the presence of anti-CD3 (0.05 ng/mL). Simultaneously, sorted CD4^+^Foxp3^+^ iTreg cells were added to the culture system at varying ratios relative to CFSE-labeled T cells. Cells were incubated at 37 °C, 5% CO₂ for 3 days. At the indicated time points, CFSE dilution, indicative of cell proliferation, was analyzed using flow cytometry (BD LSRFortessa) and quantified as the proportion of cells undergoing successive divisions.

#### RNA sequence

Total RNA was isolated from harvested cells using TRIzol reagent (invitrogen) and subsequently processed for sequencing by Beijing Novogene Bioinformatics Technology. RNA integrity and potential degradation were examined using 1% agarose gel electrophoresis, while purity was measured with a NanoPhotometer spectrophotometer. The RNA concentration and integrity were further evaluated using the RNA Nano 6000 Assay Kit on an Agilent 2100 Bioanalyzer. Library preparation was carried out with the NEBNext Ultra RNA Library Prep Kit following standard protocols, and PCR-amplified libraries were purified using the AMPure XP system. Library quality was assessed on an Agilent 2100 Bioanalyzer before sequencing on the Illumina HiSeq 2000 platform, generating high-quality 125-bp/150-bp paired-end reads for downstream analysis. Raw and processed RNA-seq data have been deposited in GEO (GSE316633).

## Statistics

Information regarding sample sizes, the number of mice per group, replicates, and statistical tests can be found in the figure legends. Sample sizes were chosen based on prior studies to ensure adequate statistical power while accounting for biological variability. Specific sample sizes are detailed in the corresponding figure legends. Unless otherwise stated, all experiments were independently repeated at least twice**.** Statistical analyses were conducted using GraphPad Prism 9, with normality tests performed to select the appropriate statistical methods. All data conformed to the assumptions required for the statistical tests applied.

## Supplementary Information

Additional file1 (PDF 3236 KB)

Additional file2 (XLSX 19 KB)

## Declarations

### Availability of data and material

The datasets used and/or analyzed during the current study are available from the corresponding author on reasonable request.
