# Supplementary material for: TGF-β signaling mediates crosstalk between CD8+ T cells and CD39+ induced Treg cells in autoimmune inflammation
Source: J Biomed Sci. 2026 Feb 24;33:21. doi: 10.1186/s12929-026-01228-z (PMC12933926; doi:10.1186/s12929-026-01228-z)
Supplement: Supplementary file 1 — Additional file1 (PDF 3236 KB) [file 12929_2026_1228_MOESM1_ESM.pdf]

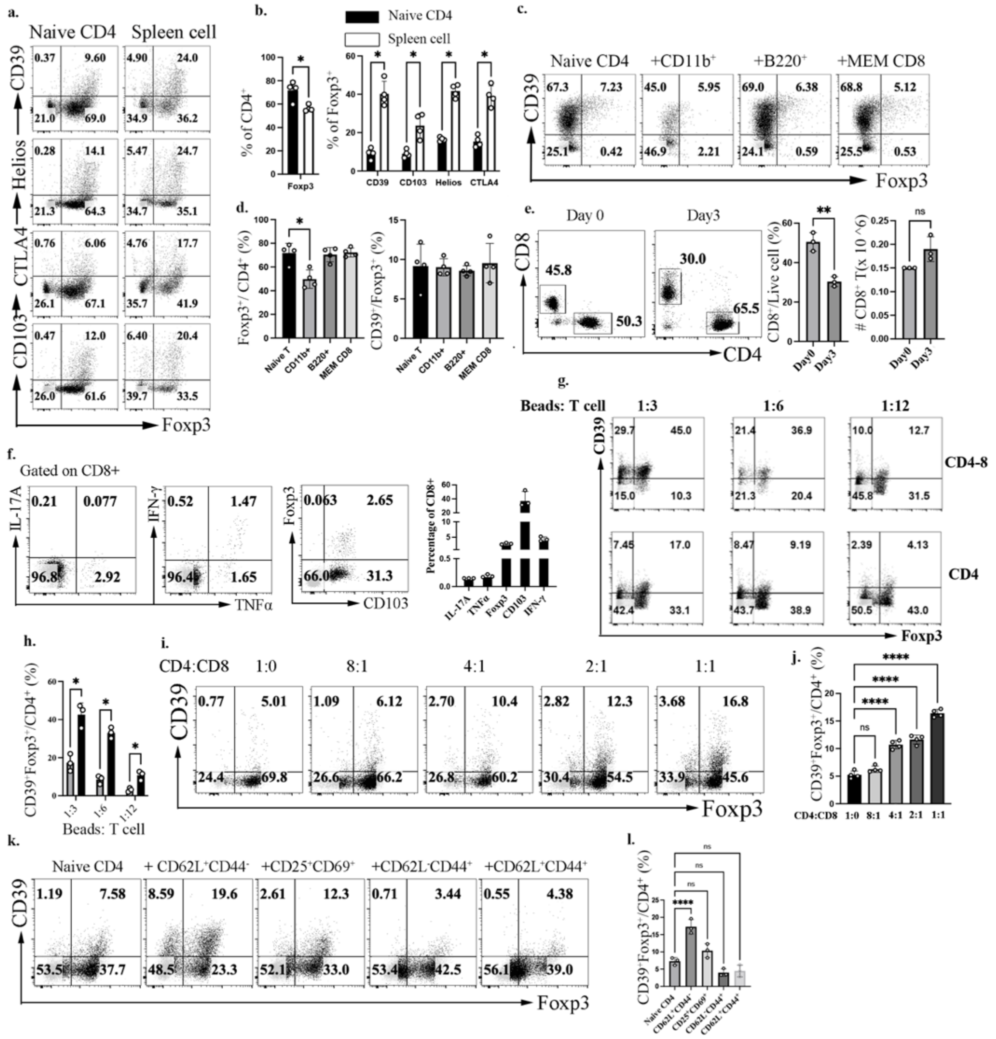

Fig. S1.

(a, b) Naïve $CD4^+$ T and Foxp3-GFP^- spleen cells were isolated and polarized under iTreg condition, flow cytometry was used to determine the expression of Foxp3, Helios, CTLA4, CD39 and CD103. Representative flow cytometry plots(a) and quantification(b) are shown.

(c, d) Sorted naïve $CD4^+$ T cells were co-cultured with other cell subsets as indicated under iTreg induction conditions. The levels of CD39 and Foxp3 were quantified using flow cytometry, the flow data (c) and the statistical data (d) were presented.

(e) Representative plots (left) and quantification (right) showing changes in $CD4^+$ and $CD8^+$ T cell frequencies and absolute $CD8^+$ T cell numbers at day 0 and day 3 of co-culture.

(f) Cytokine and phenotypic analysis of $CD8^+$ T cells following co-culture.

(g, h) Representative flow cytometry plots showing CD39 and Foxp3 expression in iTregs induced using different bead:T cell ratios(g), with corresponding quantification(h).

(i,j) Representative flow cytometry plots(i) and quantification(j) showing CD39 and Foxp3 expression in iTregs induced at different CD4:CD8 ratios.

(k,l) Representative flow cytometry plots showing CD39 and Foxp3 expression in iTregs induced from naïve $CD4^+$ T cells co-cultured with $CD8^+$ T cell subsets defined by canonical activation and differentiation markers, including $CD62L^+CD44^-$ (naïve), $CD25^+CD69^+$ (recently activated), $CD62L^-CD44^+$ (effector), and $CD62L^+CD44^+$ (memory-like)(k), with corresponding quantification.

Data are shown as mean ± s.e.m. p values were calculated by Students' t test(b,e,h) or one-way ANOVA (j,l) with by Tukey's multiple comparisons test. *$p< 0.05$, ns, not significant

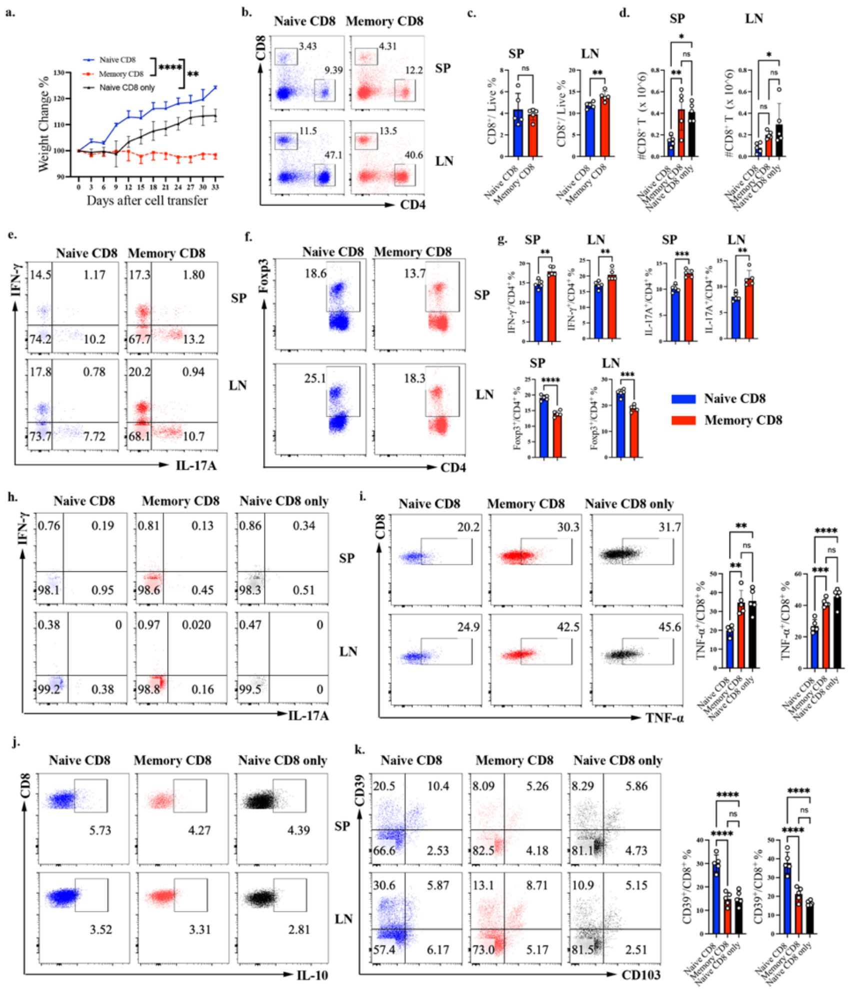

Figure S2.

(a) Body weight changes of *Rag1*$^{-/-}$ mice co-transferred with naïve CD4^{+} T cells together with either naïve CD8^{+} or memory CD8^{+} T cells over the course of disease development.
(b) Representative flow data showing CD4^{+} and CD8^{+} T-cell frequencies in the spleen (SP) and mesenteric lymph nodes (LN) at the disease endpoint.
(c, d) Frequencies (c) and absolute numbers (d) of CD4^{+} and CD8^{+} T cells.
(e) Representative data showing Th1 (IFN-γ^{+}) and Th17 (IL-17A^{+}) cells among CD4^{+} T cells.
(f) Representative flow data showing Foxp3^{+} regulatory T cells among CD4^{+} T cells.
(g) Quantification of Th1, Th17, and Treg among CD4^{+} T cells in the SP and LN.
(h) Representative data showing IFN-γ^{+} and IL-17A^{+} cells in CD8^{+} T cells in the SP and LN.
(i) Representative data and quantification of TNF-α among CD8^{+} T cells in the SP and LN.
(j) Representative data showing IL-10 expression among CD8^{+} T cells in the SP and LN.
(k) Representative data and quantification of CD39 and CD103 among CD8^{+} T cells in the SP and LN.

Data are presented as mean ± s.e.m. Statistical significance was determined by unpaired two-tailed Student's *t*-test or two-way ANOVA with multiple comparisons. *P* values are indicated in the figure.

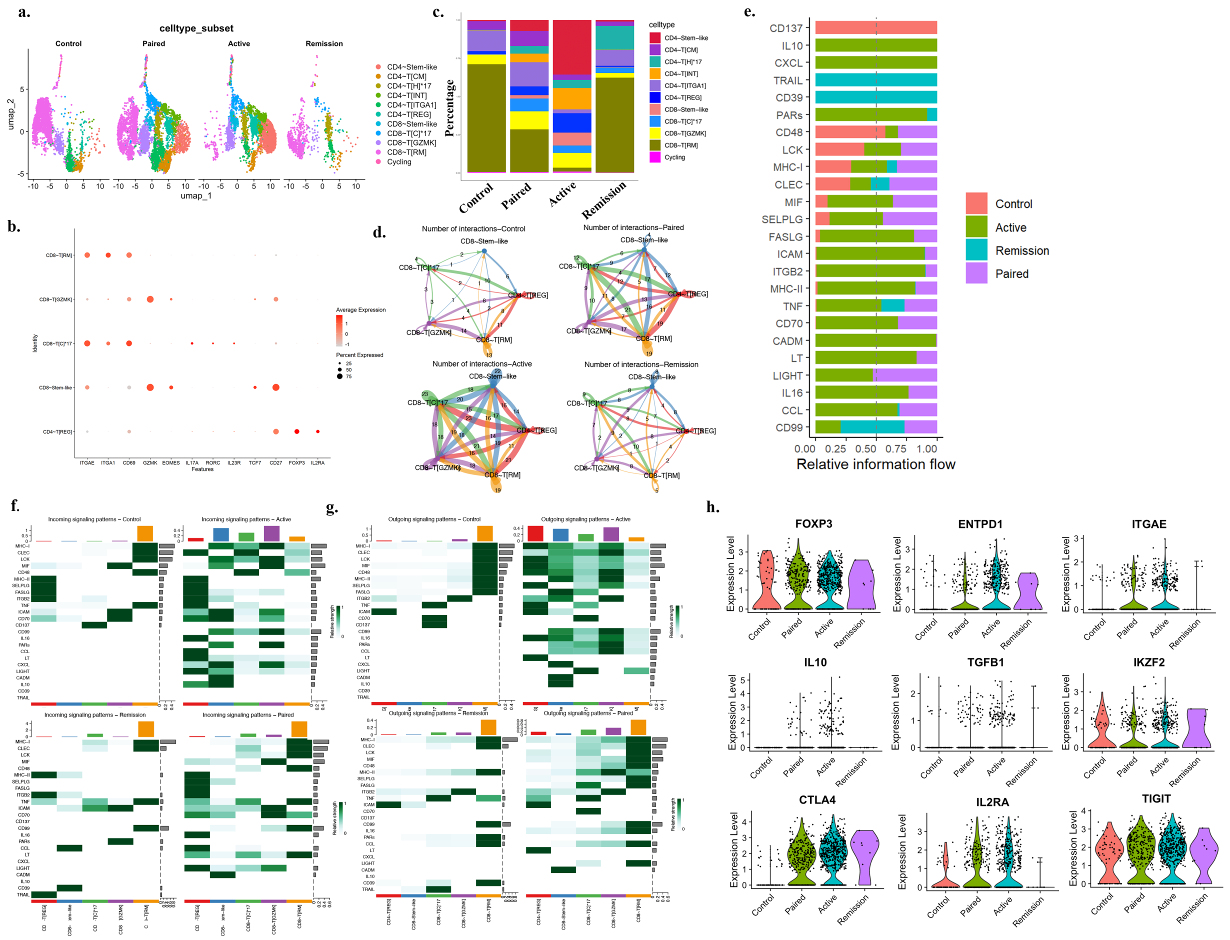

Fig. S3.

Single-cell transcriptomes of colonic T cells from healthy controls (Control) and from patients with UC at different stages, including the active stage (Active), paired healthy tissue from active patients (Paired), and remission (Remission).

(a) . A UMAP plot of 13,887 T cells clustered using Seurat is shown, with different colors representing distinct cell subpopulations.

(b) . Characteristic marker genes of each subpopulation.

(c) . Proportion of each subpopulation.

(d) . Number of interactions between $CD8^+$ subpopulations and Treg cells.

(e) . Relative intensity of key signaling pathways involved in these interactions across different disease stages.

(f, g). Incoming and outgoing signaling pathways between $CD8^+$ subpopulations and Tregs.

(h). Characteristic marker genes expressed by Tregs.
